# The Robert Wood Johnson Nurse Faculty Scholars Diversity and Inclusion Research

**DOI:** 10.1089/heq.2019.0026

**Published:** 2019-06-24

**Authors:** Versie Johnson-Mallard, Randy Jones, Maren Coffman, Jacinta Gauda, Katie Deming, Mario Pacheco, Jacquelyn Campbell

**Affiliations:** ^1^ University of Florida, College of Nursing, Gainesville, Florida.; ^2^ University of Virginia School of Nursing, Charlottesville, Virginia.; ^3^ University of North Carolina, School of Nursing, Charlotte, North Carolina.; ^4^ The Gauda Group at Grayling, New York, New York.; ^5^ Urban Institute, Washington, District of Columbia.; ^6^ Christus St. Vincent Health System, Pojoaque, New Mexico.; ^7^ Johns Hopkins University, School of Nursing, Baltimore, Maryland.

**Keywords:** bias, diversity, academics, training, scholars, underrepresented

## Abstract

**Purpose:** The purpose of this research is to deepen the understanding of DEI training and show how scholars across the nation incorporated DEI leadership into academic roles. Faculty and administrators' experiential experience in diversity, equity, and inclusion (DEI) plays a role in the success or failure of DEI training. DEI training at institutes of higher learning should include metrics that examine our bias for invisible and overt support for DEI.

**Methods:** Robert Wood Johnson Foundation Nurse Faculty Scholars (RWJFNFS) were surveyed by The Gauda Group at Grayling. Data were collected from a diverse group of scholars across the nation. An online survey followed by an in-depth phone interview was used to assess participants' roles as leaders in academic nursing, challenges faced by scholars in addressing DEI, and perceived values of undertaking DEI activities.

**Results:** Major themes emerged from the findings. The themes included championing for DEI comes with a personal and professional risk. Greater success was noted when DEI was supported by leadership and included in institutional strategic planning.

**Conclusion:** DEI is important and necessitates commitment from all levels of leadership, faculty, and strategic planning initiatives. DEI training fills an important role and subsidizes leadership effectiveness as it relates to DEI.

## Introduction

There is a distinct need for diversity, equity, and inclusion (DEI) training at institutes of higher learning for faculty, administrators, and staff. Never has this been clearer than in 2018, when a number of national events brought the issue to the forefront. The events occurred within institutions of higher learning (Yale dormitory, black student sleeping in her dormitory common area), coffee shops (Starbucks staff called the police on black men waiting for a friend to join them), television networks (ABC's the Roseanne show), and retailers (Nordstrom Rack, shopping while black). Buzz created by these events illustrated a growing awareness of the problem. Actions taken were swift and severe. Starbucks' management offered an apology and immediately assessed employee knowledge about DEI, developed diversity curriculum, and implemented training. ABC network quickly canceled the Roseanne show in response to the sitcom star's racist rant and expressed a zero tolerance policy. Nordstrom offered public an apology to the falsely accused teenagers.

As our society grows increasingly diverse, it is important that each of us examines our biases and how they contribute to hostilities that sometimes boil over into major news stories.^1,2^ Getting ahead of the problem has never been more critical at any point in history. Health care providers, such as nurses, can play an important role. Charged with providing health care to all regardless of ethnicity, age, or obesity status, nurses are on the frontline of an evolving diverse society. Nurse educators must train the next wave of nurses to embrace diverse population they serve and to set an example of inclusion for the rest of the nation.

Diversity among the nursing populations is critical, to reflect the society nurses serve. In 2017, only 19.5% of all registered nurses in the United States identified as racial/ethnic minorities.^2,3^ Medical schools continue to struggle with increasing and retaining minority students and faculty.^4^ Consistent with recent findings cited in *Harvard Business Review*, educating and working side by side with individuals from different backgrounds is essential to understanding underrepresented groups.^5^ The article found that DEI training had a positive impact in three areas:

- Engaging in problem solving
- Exposure to people from different groups
- Encouraging social accountability for change

The pivotal 2011 Institute of Medicine report *The Future of Nursing: Leading Change, Advancing Health* called on nurse leaders to focus on diversity and develop models to promote respect for race, ethnicity, geography, background, and personal experience.^6^ In 2016, the National League of Nursing encouraged nursing administrators and faculty members to achieve excellence in diversity and inclusion in student and faculty populations, workforce, and health care systems.^7^ However, in 2018 the number of Registered Nurses obtaining graduate degrees and sought roles as academicians remained small. The current study is a summary of nursing scholars' expressed thoughts and perceptions of DEI during their tenure as RWJFNFS. The purpose of this research is to deepen the understanding of how scholars perceive DEI training and how they incorporate DEI leadership in their academic lives as professors, researchers, and administrators within schools of nursing across the nation.

## Background

From 2008 to 2017, the Robert Wood Johnson Foundation Nurse Faculty Scholars (RWJFNFS) program developed the next generation of national leaders in academic nursing through career development awards for outstanding junior nursing faculty. The program aimed to strengthen the academic productivity and overall excellence of nursing schools by providing mentorship, leadership training, salaries, and research support to enhance scholars' communication and leadership skills, and the opportunity to network with national and international leaders. The program embraced racial, ethnic, and gender diversity and encouraged applications from candidates with diverse backgrounds. Furthermore, DEI-related learning experiences were purposely included within the leadership curriculum for the scholars, and a strategic plan was developed in 2010 to increase recruitment of diverse applicants.^8^

While ethnic diversity in the U.S. population continues to grow rapidly, the health care workforce is diversifying at a much slower pace. Translating DEI-related learning experiences to the recruitment of diverse applicants is a next step in hiring and retaining minorities in the health care workforce. DEI training provides support for those championing diversity—a stronger business case, a sense of support and community, the ability to leverage initiatives, and shared learning. A strong, resourced diversity plan that includes buy in at the administrative level demonstrates that diversity is a priority.^9^ Evidence of such an initiative might include having a diversity officer in a high-level administrative position and including diversity initiatives in the strategic plan,^1,10^ or developing a taskforce to measure DEI outcomes.^5,11^

Intensive faculty development/mentoring programs can improve the success and retention of diverse faculty members.^10,12^ The most effective programs use a multifaceted approach. One such program aimed to change the faculty culture in medical schools through a program that included 14 sessions over 2 years and used focused dialog, self-reflection, skill building, and collaborative approaches.^13^ The Nursing Faculty Scholars program intentionally used a diversity plan to increase the number of applicants from underrepresented groups, and incorporated DEI content into the curriculum throughout the 3-year program.^8^

Cultivating and translating institutional DEI training around racial, ethnic, and social economic is limited in published research. DEI strategic planning was identified by national nursing and medical academic units as a great need. DEI training promotes faculty development and health equity when measurable outcomes are supported. Nurse scholars of the RWJF were exposed to experiential DEI training through collaborative learning. This article is offered to proliferate the understanding of how scholars incorporate DEI training into their professional roles.

## Methods and Procedures

The goal was to obtain 20 or more scholars from the NFS cohorts from 2008 to 2013 and have representation from each cohort to get a better understanding of the scholars' leadership develop experiences. The Gauda Group at Grayling was commissioned to design an online survey, to contact RWJFNFS, conduct interviews, and analyze the results. All cohorts were represented in the final sample, except for the 2010 cohort, which did not have any underrepresented scholars. Of the 27 scholars who participated, 42.3% (*n*=12) of the scholars self-reported as being from an underrepresented minority group, 81.5% (*n*=22) were Associate Professors, and 26.92% (*n*=7) were male. See Table 1 for additional demographic data.

**Table 1.** Demographic Data

Academic rank, *n* (%)	
Assistant professor	4 (14.81)
Associate professor	22 (81.48)
I prefer not to answer	1 (3.7)
Cohort year, *n* (%)	
2008	7 (25.93)
2009	5 (18.52)
2011	3 (11.11)
2012	6 (22.22)
2013	6 (22.22)
Total	27 (100)
Race/ethnicity, *n* (%)	
White	14 (53.85)
Black or African American	4 (15.38)
Asian or Asian American	2 (7.69)
I prefer not to answer	3 (11.54)
Latin/Hispanic	1 (3.85)
Middle Eastern or North African	1 (3.85)
Multiracial	1 (3.85)
Unanswered	1 (3.84)
Gender identity, *n* (%)	
Female	17 (65.38)
Male	7 (26.92)
I prefer not to answer	2 (7.69)
Total	26 (100)
Unanswered	1

The two-part research model consisted of a 23-question online survey and a subsequent telephone interview. The online survey included aided, unaided, and open-ended questions and statements that focused on the level and nature of the program's influence on scholars from a DEI perspective. The NFS' impact on participants' roles as leaders in academic nursing, challenges faced by scholars in addressing DEI issues or participating in DEI initiatives, perceived value of undertaking DEI activities in a scholar's specific School of Nursing, and perspectives on additional research in DEI is reported.

A discussion guide for the interview segment was provided to participants before the telephone interview. The interviews generally were completed in about 30–45 min. Notes were taken. Each of the actual 12 questions are attached (Table 2). The follow-up telephone interview allowed respondents to expand on/clarify their survey responses and add additional context on their views and experiences related to DEI.

**Table 2.** Survey Questions

Q1. In the online questionnaire, we asked about the kinds of actions in support of DEI within your institution that you may have been inspired to take as a result of your NFS leadership development experience. (if you were inspired) Please describe the actions you took. Did these actions contribute to any changes in practices, policies, and programs within your institution?
Q2. When it comes to championing DEI within your institution, what would you say are the important leadership skills/competencies or qualities that the NFS experience has helped you to develop?
Q3. Becoming a leader with a strong commitment to DEI within academic nursing may require taking risks. If you agree, what are some of these risks? Did you find yourself having to take any risks? If so, please describe the kinds of risks that you have taken to support the DEI agenda within your institutions.
Q4. If time and resources were unlimited, what other learning experiences do you feel would enhance your ability to lead and or influence the advancement of DEI with your institution?
Q5. After program completion, how could or can NFS have been/or be more supportive of your efforts to lead in DEI?
Q6. What features/benefits of DEI do you or others believe should be stressed in your institution to increase an active commitment to DEI?
Q7. What criteria would you use to measure leadership effectiveness in academic nursing when it comes to DEI?
Q8. Have you ever faced resistance to your efforts to promote/advance DEI within your academic nursing career? If so, please explain.
Q9. Have your activities with respect to DEI influenced your quest for tenure or ability to achieve it? If so, please explain.
Q10. What kinds of content would you like to see provided by the NFS programs, and future scholars/fellows programs in academic nursing, regarding the issues/challenges faced by nursing faculty who are one of only a few underrepresented faculty at their institution?
Q11. What kinds of content would you like to see provided by the NFS program, and future scholars/fellows programs in academic nursing, regarding the issues/challenges faced by nursing faculty who are one of only a few underrepresented faculty at their institution?
Q12. Are there any other recommendations or comments that you would like to provide concerning how the RWJF NFS leadership development experience may have informed your leadership style, research, or other professional activities?

DEI, diversity, equity, and inclusion; RWJFNFS, Robert Wood Johnson Foundation Nurse Faculty Scholars.

### Data analysis

The surveys were transcribed, and potential identifiers were removed to maintain data confidentiality. Participants' *thick descriptions* of their experiences related to DEI were captured.^14,15^ *Thick descriptions* are defined as comprehensive descriptions of data that build an analytical explanation of what has occurred. An *iterative approach*, defined as an ongoing analysis of the data to categorize it, identified themes. The authors read the transcripts several times, coded them, identified strips (brief data extracts to capture significant participant views), and then created categories and extrapolated themes. The authors independently reviewed transcripts to validate the themes then confirmed the results among themselves creating credibility, dependability, and confirmability.^16,17^ Credibility was established by active engagement with scholars' data and narratives along with member checking. Dependability and confirmability were established with an audit trail. Authors conducted a comprehensive review to clarify scholars' experiences with DEI. This process allows for transferability of the findings.^16^

### Findings

Across the 27 transcripts, 5 major themes emerged: (1) NFS provided knowledge and understanding of DEI purposes and practices; (2) Academic Institutions need to give serious attention to DEI training; (3) DEI can be perceived as a potential risk; (4) DEI does not meet academia's requirement for progression; and (5) accurately measuring DEI leadership effectiveness is needed.

#### NFS provided knowledge and understanding of DEI

Respondents clearly perceive that the program provided the necessary knowledge and support of DEI to enhance their personal and professional development. While many indicated that they had an interest in DEI before their admission to the leadership development program (a criterion for consideration), all respondents acknowledged a deeper understanding of DEI resulting from the leadership development experience. Many reported new, changed, and enhanced insights gained from the experience. They cited three factors:

- The diverse nature of the cohorts that allowed for meaningful dialog and shared experiences
- The importance that the program leadership placed on DEI, which was clear and purposeful
- Specific training experiences that focused on sensitive topics such as white privilege

One participant stated,

The NFS experience helped make DEI issues more visible for me. It was helpful to learn from colleagues and leaders in academic nursing as well as hear about successful cases.

Another participant said,

NFS was very successful at expanding my knowledge of DEI by virtue of the diverse cohort and opportunity to interact with diverse scholars.

The majority of the participants believed that by understanding more about DEI, they were better able to recognize and act on their own biases. About 70% said the DEI experience would have an enduring, sustainable impact on their leadership. Some 81% said the leadership development experience would significantly affect their roles as leaders in academic nursing. One respondent stated,

Most important was being able to step back and assess my own biases. This helped in assessing the institutional environment and where change might be possible. The training on various institutional models of DEI was also helpful.

Another respondent said,

In the beginning of the program, we focused on different personality types. This helped with my own humility and leadership skills. It's important to work with people who approach problems differently. It allowed me to come across more inclusively. It's really more about diversity of thoughts and skills. I realized even more that there's not one right way to do or think. Also, the team building exercises affect all the work I do.

Many of the comments supported the idea that newly acquired DEI knowledge and support better prepared them for leadership. One scholar stated,

Being a leader requires you to be outspoken. You can't simply complain to someone else. You need to be able to speak out to the powers that be. You can't have the fear of retribution. You need to know your worth. If you feel worthy, you should be able to speak out on behalf of others who need your help.

Another participant said,

It was helpful when doing committee work to better understand how to communicate. I learned how to tell stories to different people, listen better, and accept people different than me. It's a good skill to be able to see other people's perspectives.

#### DEI within academic institutions

Scholars often note that having the support and commitment of the academic institution, particularly the dean, is critical to promoting and sustaining diversity efforts. The presence of DEI in the strategic plan is also valuable. One scholar noted that his school embraced diversity as part of the strategic plan, and deans consistently articulated the value of DEI. Another respondent emphasized the commitment to DEI at the university's highest levels. One scholar agreed with this commitment, saying,

We must infuse DEI initiatives into the structure (not outside of it). When I am gone, the DEI focus should not leave.

Respondents sometimes mention DEI in the context of the larger institution. Some scholars mentioned the link between the academic institution perspective on DEI and the surrounding community, as well as the need for a diverse workforce. One respondent stated,

It's critical that we [nurses and other healthcare providers] catch up with demographics and look like the communities around us. Diversity gives fresh eyes, new perspectives, and a richness to work that we need. Also, there are issues of fairness and social justice. There's a strong thread of social justice in nursing. Nurses can lead in that regard. DEI falls in line with that. DEI students and faculty make my work better. It's enriching.

Another participant stated,

We are a public institution; the community is empowering us to serve. The void of diversity is the void of richness, including intellectual richness. The more diverse we load the table, the more competitive we are. We want the nursing workforce to reflect the community they are serving, and we must reflect this within the academy.

A participant commented on the need for a more diverse academic workforce,

The profession is made up of a majority of white women. The diversity of the health workforce needs to be increased. If you increase the diversity of faculty, there's greater interest among [underrepresented] groups in becoming a future faculty member… If males see more male faculty in nursing, there will be more interest among them in nursing.

Schools of Nursing and academic institutions have a responsibility to educate and give thoughtful attention to DEI. Educating future health care providers to work beside diverse colleagues and provide equal care for a diverse community is essential.

#### DEI can be perceived as a potential risk

With a few exceptions, scholars perceived that there are risks in being a DEI champion. Scholars made direct and indirect links between DEI champion behaviors and certain unfavorable consequences—isolation, exclusion from committees, minimizing of contributions, being nonresponsive to input, being branded as “difficult,” and potentially offending colleagues or losing allies. A scholar stated,

You have to worry about offending people or losing friends. But it sometimes can't be helped. I have to look for people to work with me.

Another participant said,

I think you can be negatively affected if DEI isn't a priority at your institution… I have been personally affected. You can be ostracized and will feel tension from your colleagues. But, I have tenure, so the risks are lower.

Another participant stated,

Yes, I agree. Speaking out on behalf of marginalized groups, which include LGBTQ or people of lower academic rank than me. Pushing for hiring minority faculty. The risk is that you take an unpopular side and hurt your academic career. You could be seen in an unfavorable light by your colleagues.

Interviews with participants generally indicate that diversity-related challenges are manifested in less obvious ways. They or their colleagues are not necessarily encountering outright resistance or clear prejudice. Participants reported that when they were unable to pinpoint the exact intentions, forms of exclusion that are subtle or reside under the surface can cause distress. One person stated,

There is some contention between majority and minority faculty. It can be at the surface or in the background. At my school, there are more people from minority backgrounds now. Sometimes appreciating the effort to recruit and retain more diverse faculty may not be something that the majority faculty wants to hear. Some people in the majority may feel threatened. Talking about DEI can increase contention, so you have to be savvy when discussing it… Conversations about unconscious bias can create unease and bad feelings. The attitude among some in the majority may be along the lines of “Are you accusing us?”

There are perceived risks in bringing DEI ideas to colleagues and institutions. These perceptions may hinder creative thought, appropriate and needed institutional changes, and the ability to have an open dialog to enhance personal and academic productivity.

#### DEI is not recognized to meet academia's requirement for progression

Being a DEI champion offers moderate rewards for scholars because DEI achievements are seen as a value add after established requirements are met. Nearly half (48%) of the scholars believed that DEI activities and research were rewarded and/or recognized. Fewer (37%) said that DEI was somewhat rewarded and/or recognized. As one respondent stated,

I don't really know how they came to the decision to award me tenure. I think [DEI] did help to some extent. My mentorship of students and interest in the health of minority populations probably helped. I highlighted my interest in the health of vulnerable populations in my application for tenure.

Another participant stated,

I did have challenges with the [minority health-related] research, so it probably limited my ability to publish more. However, the research I did was important and showed hard work. So, it probably balanced out.

Research/scholarship is more highly valued in the promotion/tenure processes than DEI activities, according to participants. They often stated that DEI was categorized as a service and not a credential. One participant said,

DEI activities are lumped with the service work category. I'm with a Research One institution, so research is very important, probably 99.5% of how you are evaluated. As long as you're a passable teacher, teaching isn't weighted that much. Service is the frosting on top. You have to do it, but it doesn't weigh heavily… If you get people riled up with you, you may not get tenure. You need to be diplomatic and thoughtful about the issues you take on and how you handle them.

Many respondents said their academic institutions did not incorporate DEI activities in their promotion/tenure decisions. This was particularly true for research-intensive institutions.

#### Accurately measuring DEI leadership effectiveness

Accurately measuring DEI success and evaluating leadership effectiveness in academic nursing is needed. Respondents often mentioned the importance of quantitative metrics to determine the effectiveness of efforts to increase diversity in hiring or DEI leadership. A common measure that respondents stated that could be used was examining recruitment and retention of diverse faculty. One respondent stated,

Hiring diverse faculty…. You can look at how many diverse faculty there were before the Dean came in and then after. So, recruit and retain diverse faculty.

Another participant stated,

You need to measure the impact of the program. You also need goals. So, for example, shoot for 40% of 50 students at an institution being part of the minority community.

Going beyond overall percentages of diverse faculty and staff, a number of respondents said there should be a focus on the progress and achievements of minority students and faculty. In particular, are minority faculty and students engaging in DEI activities? One respondent stated,

Assess strategies developed to promote DEI. What are the leader's visions for obtaining resources, deploying the right people for DEI-related tasks, and developing a welcoming and inclusive environment? Does the leader focus just on putting out fires or is there a larger plan over 5 or 10 years for promoting DEI in the institution and community?

Another participant stated,

You can monitor it by making DEI a component of annual reviews and merit increases. So, the question for faculty and staff would be: What did you do to change DEI?

Respondents emphasized that measuring DEI more accurately may help to assess leadership and offered the example of whether a faculty member feels like he or she is part of the team. This also translates to the impact that an institution's administration or dean may have toward a vision for DEI if it is considered a priority.

## Discussion

Being a champion for change within an established institution involves varying degrees of risk taking. Change agents championing DEI need knowledge of their institutions' vision and goals regarding diversity. They need a commitment from leadership, sponsors for DEI-related endeavors, and access to resources for successful change. DEI champions are aided by the ability to garner support and navigate change and to frame DEI discussions. He or she must anticipate reluctance and/or resistance, and personally be open to differing perspectives and ideologies.^5,11^

Many participants reported that they have witnessed or experienced negative consequences in response to their DEI efforts. While many persisted in their efforts, the perceived risks they took suggests the need for a skillful approach to DEI within schools of nursing. Most scholars (particularly from the majority group) reported that the program had given them new and/or enhanced insights due to interaction, shared experiences, and listening to firsthand accounts from diverse participants. This finding is supported in the DEI literature.^5,10,18^

Interviews with scholars confirmed the idea that committed leadership is fundamental in sustaining DEI. This level of commitment ensures that DEI is embedded in the institution's strategic plan. It boosts the confidence of individuals who are not in leadership positions but able to influence change. Scholars indicated that DEI training fills an important role by continuing to contribute to the body of knowledge about leadership effectiveness as it relates to diversity.

## Abbreviations Used

DEI: diversity, equity, and inclusion
RWJFNFS: Robert Wood Johnson Foundation Nurse Faculty Scholars
